# fMRI measurements of amygdala activation are confounded by stimulus correlated signal fluctuation in nearby veins draining distant brain regions

**DOI:** 10.1038/srep10499

**Published:** 2015-05-21

**Authors:** Roland N. Boubela, Klaudius Kalcher, Wolfgang Huf, Eva-Maria Seidel, Birgit Derntl, Lukas Pezawas, Christian Našel, Ewald Moser

**Affiliations:** 1 Center for Medical Physics and Biomedical Engineering, Medical University of Vienna, Vienna, Austria; 2 MR Centre of Excellence, Medical University of Vienna, Vienna, Austria; 3 Social, Cognitive and Affective Neuroscience Unit, Department of Basic Psychological Research and Research Methods, Faculty of Psychology, University of Vienna, Vienna, Austria; 4 Department of Psychiatry, Psychotherapy and Psychosomatics, RWTH Aachen University, *Aachen*, Germany; 5 Department of Psychiatry and Psychotherapy, Medical University of Vienna, Vienna, Austria; 6 Department of Radiology, Tulln Hospital, Karl Landsteiner University of Health Sciences, Tulln, Austria; 7 Brain Behaviour Laboratory, Department of Psychiatry, University of Pennsylvania Medical Center, Philadelphia, PA, USA

## Abstract

Imaging the amygdala with functional MRI is confounded by multiple averse factors, notably signal dropouts due to magnetic inhomogeneity and low signal-to-noise ratio, making it difficult to obtain consistent activation patterns in this region. However, even when consistent signal changes are identified, they are likely to be due to nearby vessels, most notably the basal vein of rosenthal (BVR). Using an accelerated fMRI sequence with a high temporal resolution (TR = 333 ms) combined with susceptibility-weighted imaging, we show how signal changes in the amygdala region can be related to a venous origin. This finding is confirmed here in both a conventional fMRI dataset (TR = 2000 ms) as well as in information of meta-analyses, implying that “amygdala activations” reported in typical fMRI studies are likely confounded by signals originating in the BVR rather than in the amygdala itself, thus raising concerns about many conclusions on the functioning of the amygdala that rely on fMRI evidence alone.

The human amygdala is the target of a large number of imaging studies due to its central role in emotion processing^1^,^2^, emotional learning^3^ and its potential involvement in various psychiatric disorders^4^,^5^,^6^. Functional magnetic resonance imaging (fMRI) in particular is one of the tools most commonly employed to study the role of this brain region^7^, and indeed has proven a valuable resource (at the time of writing, a pubmed search using the search terms ‘(“fmri” or “functional magnetic resonance imaging”) and amygdala’ yields 2500 results). Still, there is notable heterogeneity and disagreement between fMRI studies of the amygdala, both in terms of activations in tasks^8^ and functional connectivity during rest^9^.

Typical forms of disagreement between studies are the failure of newer studies to replicate results from earlier papers or to find any significant results in the amygdala at all^9^. More subtle effects can be differences in lateralization between studies^10^ or unintuitive laterlization effects within a study. For example, Manuck *et al*.^11^ suggest that their “observed right laterality bias reflects the visuospatial processing demands of [their] paradigm, which preferentially engages right hemisphere circuits”, but without explaning the exact mechanisms that would cause this effect on their measured amygdala activations.

Hints for the difficulties in replicating previous amygdala fMRI results might be found in the outcome of reproducibility studies of activation patterns in the amygdala^8,11–13^. These studies found that among the paradigms and regions of interest studied, the amygdala activation is the least reproducible both at group and at single-subject level^8^,^13^, and that reproducibility of amygdala results even decreased after physiological noise correction, suggesting that the most reproducible findings in the amygdala region might be due to physiological effects^8^. In addition, repeatability was much lower at single-subject level than at group level, leading to a critical view on potential diagnostic uses of such data as opposed to group-level comparisons only^8^,^13^.

Another contribution to the heterogeneity of fMRI results might be found in the type of stimuli used. Emotional faces are a typical cue to evoke amygdala activations, but difficulties arise when choosing an appropriate control condition: neutral faces are considered unreliable in this respect^12^, so non-face control stimuli are more widely used as control condition, but bear the risk of mixing activations due to emotion with activations related to face recognition. More generally, in an early fMRI meta-analysis, Phan *et al*.^14^ found that visual stimulation is more robust in inducing amygdala activation than auditory stimulation (note that none of the studies using auditory cues lead to activations in the amygdala), and that fear is the most robust emotion to evoke activation, with a much higher proportion of studies using fearful emotional cues yielding significant results in the amygdala than happiness, sadness, anger or disgust. This difference in amygdala activation strength depending on the emotion expressed by the faces shown is corroborated by a later meta-analysis by Fusar-Poli *et al*.^15^. More recent studies though found amygdala activations also with non-visual stimuli, including increased amygdala activation in reaction to auditory stimuli in blind subjects compared to healthy controls^16^. Amygdala activations in response to emotion can be evoked in a wide variety of different ways, as shown by various studies employing different paradigms, including auditory, haptic and even intrinsic (e.g., memory recollection) stimuli. Meta-analyses investigating the results of these studies have identified significant variability across different stimulation types, highlighting potential heterogeneity in amygdala activation patterns introduced by the paradigm design. There exists evidence that visual stimuli are among the most robust in producing amygdala activations^17^, with a meta-analysis on subliminal stimuli pointing into the direction that in this particular case, reproducible activations in the amygdala region could be found only in visual stimulation using faces, not with any of the other (somatic, auditory, lexical) paradigm types^18^. Still, even among visual stimuli, the most commonly employed type of paradigm, there is considerable variability in terms of the exact setup of the paradigm as well as in the results induced in terms of amygdala activation. The origin of heterogeneity across different types of paradigms is not yet understood, and it is unclear whether it is due to different responsivity of the amygdala to different types of stimuli or to confounding factors introduced by different types of stimulation that are not directly related to amygdala responsivity.

Another issue that has been investigated as a potential source of inconsistencies is low signal-to-noise-ratio (SNR), which can be problematic in the amygdala region due to local magnetic field inhomogeneity^19^,^20^. Indeed, time series SNR is low in many voxels in and around the amygdala and can vary greatly between left and right amygdala^12^, an observation which suggests that researchers should be wary of null findings in these areas, as they might rather reflect signal loss than absence of neuronal activity, in particular in medial and ventral parts of the amygdalae where signal dropout is greatest. Moreover, lateralization effects are only rarely tested for statistical significance and thus often represent only small, statistically insignificant differences unlikely to be replicated in later studies. For the same reason, even when a study reports amygdala activations to be significant only in one hemisphere, the *difference* between the left and right amygdala activations might in itself not necessarily be statistically significant, and such a result should therefore not be misinterpreted as evidence in terms of lateralization effects. While this is true both in this particular case as well as in general when interpreting null results of statistical tests, it does not preclude the investigation of other leads concerning the reasons of the unreliability of results. Of note, Johnstone *et al*.^12^ achieved fairly good SNR values across the whole amygdala, but nonetheless the activation patterns shown spanned primarily the dorsal and medial parts of the amygdalae, which in their case is less likely to be due to SNR issues in the more ventral parts. Additionally, they found higher reproducibility in the left amygdala than in the right one, despite the SNR values for the left amygdala being lower—hinting at the idea that SNR might not be the only issue at work here.

New MR sequences might help to shed light on the constitution of signal variability in the amygdala region. Multiband Echo-Planar Imaging (EPI) sequences^21^ allow for the acquisition of fMRI time series with very high temporal resolution that significantly increase functional SNR^22^,^23^ and are able to critically sample physiological high-frequency fluctuations, thus offering the possibility to distinguish between these signals and low-frequency fluctuations in areas where both types are abundant, such as the amygdala region^24^. Indeed, overcoming the reduction in SNR due to aliased high-frequency oscillations as well as a higher sampling of the signal variation in its own right leads to a better understanding of the oscillations in and around the amygdalae. Thus, in the present study, we investigated activations in the amygdala region to the presentation of emotional faces using these new techniques alongside more conventional Blood-Oxygenation-Level-Dependent (BOLD) EPI sequences to investigate the origins of signal fluctuations and their heterogeneity in this region and the effects this may have on fMRI research using standard scanning techniques.

## Results

We used three main approaches to address the question stated above. The main body of evidence is a low-TR multiband EPI dataset of 16 subjects, comprising both a typical amygdala activation task and a resting state scan, supported by susceptibility-weighted imaging (SWI) for the identification of cerebral veins (in addition to standard T1-weighted reference images). To show that results also apply to standard fMRI studies and rule out the possibility that findings in the first dataset are merely artifacts of this new acquisition method and/or its scan parameters, a comparison dataset of 134 conventional high-TR BOLD EPI scans is used where the same amygdala activation task is employed. Finally, results are complemented by a comparison of the maps identified here with activation cluster coordinates from the literature to underline that distorted results due to the effect described here are indeed widespread.

**Figure 1:**
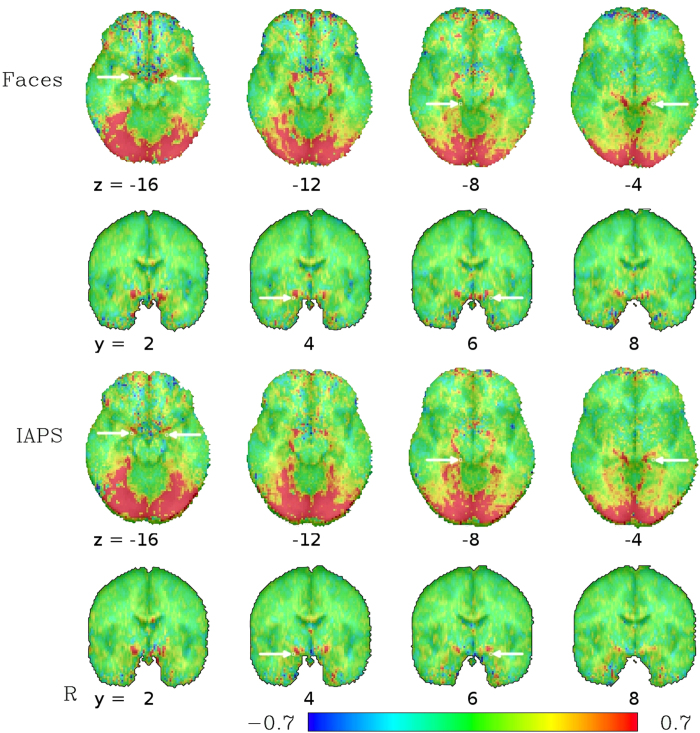
Average functional activation from 16 subjects measured with a low-TR (333 ms) multiband EPI sequence for the contrast between fearful faces (top) and threatening IAPS pictures (bottom) compared to geometric forms in a block-design matching task. Note the activation pattern in the amygdala area following the typical course of the basal vein of Rosenthal (BVR) around the brainstem until no longer distinguishable from the activation cluster in the occipital lobe. The values depicted are beta coefficients for the linear models averaged across subjects and can be interpreted as percent signal change between the faces/IAPS blocks on one hand and the geometric figures on the other. Arrows indicate locations of activation foci in the meta-analysis by Sabatinelli *et al*.^27^, the arrows in the axial slice at z = −16 and in the coronal slices at y = 4 and y = 6 pointing to the amygdala foci, the arrows in the axial slices at z = −8 and z = −4 pointing to the parahippocampal gyrus foci, see table 1. Note the close proximity in particular in the amygdala region between the meta-analytic activation foci and the BVR activations.

**Figure 2:**
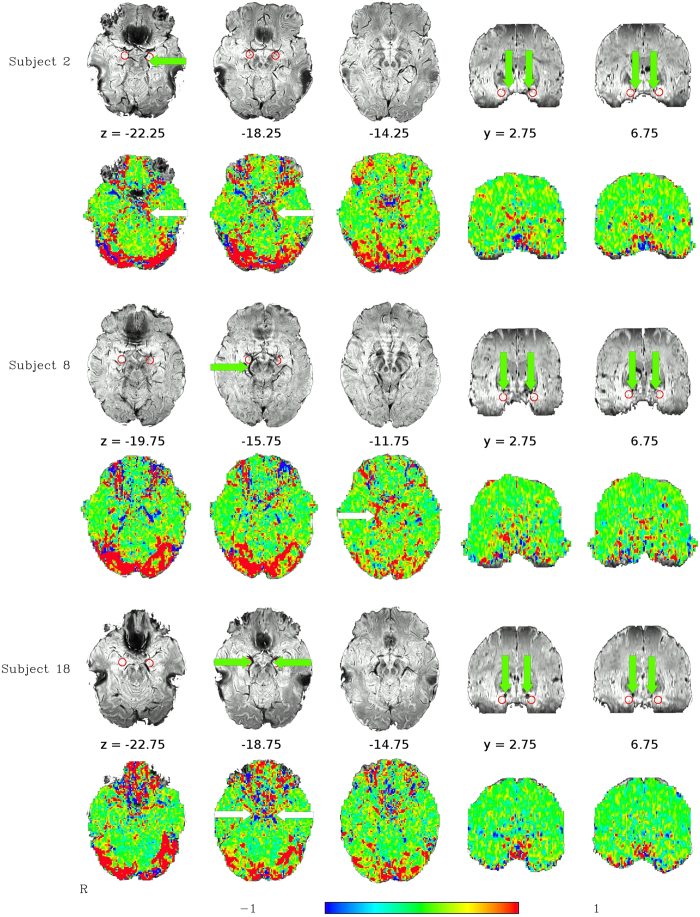
Example single-subject susceptibility-weighted images and results from the GLM of the matching paradigm (using the contrast ‘Faces – Forms’). Subject 2 exhibits a clear posterior drainage of the BVR (green and white arrows) around the brain stem in the left hemisphere, subject 8 in the right hemisphere, and subject 18 in both. Despite some geometric distortions between the two images, leading to the vein not always appearing on the same slice in the two, a correspondence between the activations and the veins in the SWI images (black) is visible. Approximate amygdala positions for each subject encircled in red.

**Figure 3:**
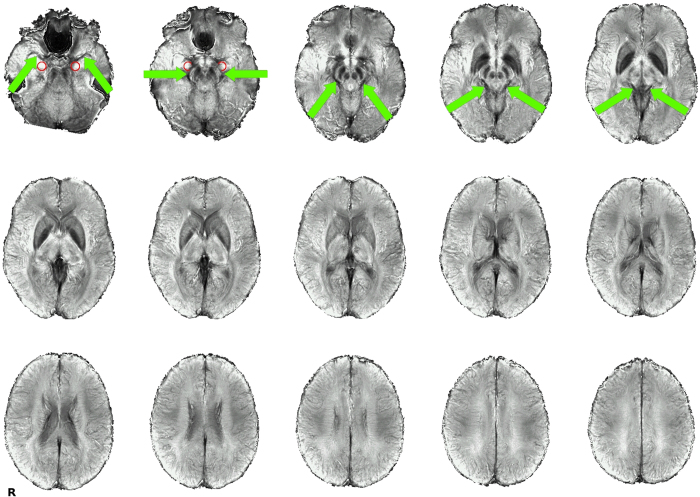
Mean SWI. Though most smaller brain vessels vanish when averaging SWI images across subjects, the typical course of the BVR is clearly visible as a dark line from the uncus close to the amygdala, around the brain stem to the vein of Galen. The course of the BVR is highlighted with green arrows. Approximate amygdala position encircled in red.

The analysis of the low-TR datasets, after standard preprocessing except for the omission of spatial blurring, yielded group-level activation maps for the contrasts ‘Faces − Forms’ with bilateral activation peaks in the occipital lobe, fusiform gyrus, middle frontal gyrus as well as in the amygdala region and around the brain stem (see Fig. 1). Activations for the contrast ‘IAPS − Forms’ were essentially in the same regions, with very similar spatial activation patterns. The activation in the amygdala region is particularly noteworthy insofar as it does not represent a focal activation cluster centered on the amygdala, but rather follows a linear course from the amygdala around the brainstem until it joins the posterior activation cluster in the occipital lobe. This corresponds to a typical course of the basal vein of Rosenthal (BVR)^25^,^26^, which can be identified in the single-subject SWI data. Indeed, for the 13 subjects for which SWI data were available, this course of the BVR with a posterior drainage could be observed in 16 hemispheres (6 hemispheres showed a clearly different path of the BVR, 4 could not be clearly identified). These single-subject SWI datasets can be compared to the single-subject activation maps from the matching task to identify a correspondence between the venous path in the SWI datasets and the activation clusters in the amygdala region (see Fig. 2). Since the vein is in some cases difficult to track on the limited number of slices displayed in a figure on paper, we have also compiled a video scrolling through all the relevant slices for all subjects (see supplementary video 1). Despite anatomical variability across subjects, the typical course of the BVR can even be distinguished on a mean SWI image averaged across all subjects (see Fig. 3).

**Figure 4:**
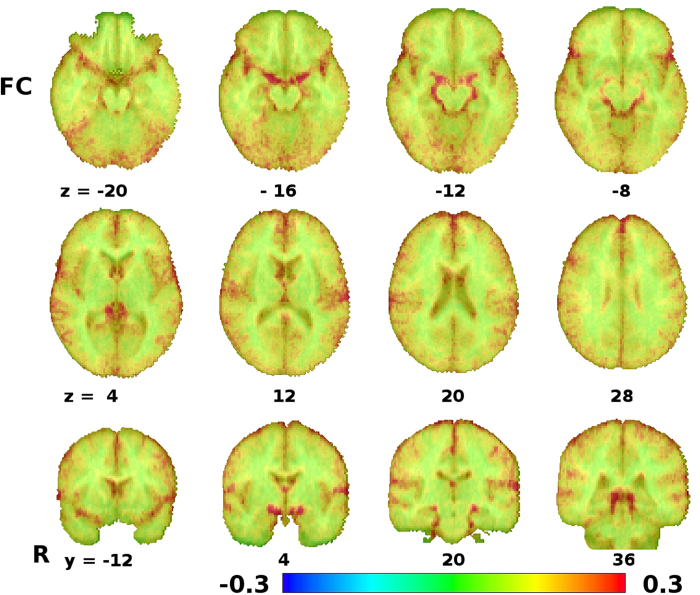
Resting-state functional connectivity from 16 subjects measured with a low-TR (333 ms) multiband EPI sequence using each subjects 100 most strongly activated voxels in the amygdala region as a seed at single-subject level, r-to-z transformed and averaged across subjects. Signals in these voxels are most strongly correlated with voxels containing large vessels, as around the brain stem and in the lateral fissure.

**Figure 5:**
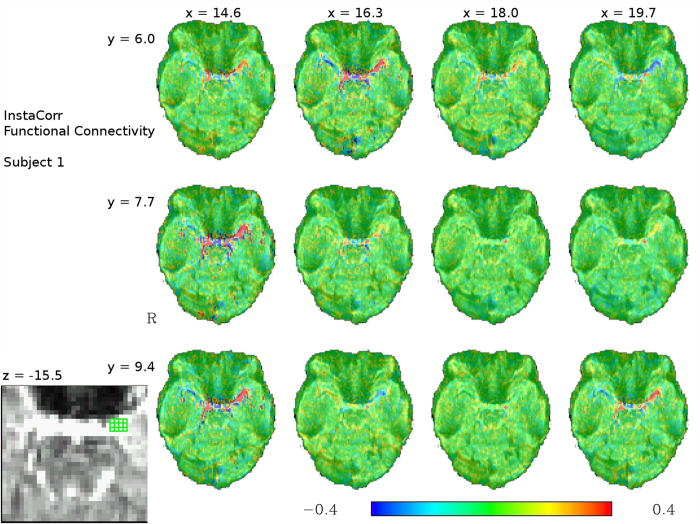
InstaCorr functional connectivity maps for 12 voxels at the amygdala/BVR border for one exemplary subject from the low-TR (333 ms) dataset, with colors representing correlation coefficients. The location of the 12 seed voxels are shown in the bottom left corner in a zoomed-in view of the raw EPI slice. Seeds in the BVR, e.g. at (14.6, 7.7, 15.5), have a characteristic connectivity pattern involving strong correlations to other voxels in the vasculature, whereas seeds more clearly within the amygdala itself, e.g. (18, 7.7, 15.5), display no correlation with these voxels. The top right seed voxel at coordinates (19.7, 6, 15.5) corresponds to the left amygdala activation focus in Sabatinelli *et al*.^27^ and shows an intermediary pattern in this particular subject, with apparently some contamination by venous signal.

To assess whether the activated voxels were more likely to reflect signal changes within the amygdala or in the vasculature around it, resting-state functional connectivity was computed from those same voxels, using the voxels with highest activations from the matching dataset in the amygdala region as seed regions for each subject. Rather than using a thresholded activation map directly, which leads to seed regions of different sizes across subjects and therefore might introduce some bias, we opted to use each subject’s 100 voxels with the highest t-values in the amygdala region as the seed for the functional connectivity analysis. Mean functional connectivity z-scores for 16 subjects are shown in Fig. 4, revealing a high correlation of the signal from the voxels in the amygdala region that were activated in the task with further voxels on the path of the BVR, as well as other regions characterized by the proximity of large vessels, in particular the lateral sulcus. This pattern of connectivity is markedly different than the functional connectivity associated with the amygdala in previous studies, as well as the functional connectivity of voxels more clearly within the amygdala in our dataset, as identified from various different voxels in the amygdala, but further away from the BVR at its border using AFNI InstaCorr (see Fig. 5). This means that the peak activation voxels in the task GLM are, considering their functional connectivity structure, more likely to be located in the BVR than in the amygdala itself. Note also that functional connectivity patterns can vary greatly between even neighbouring voxels in the amygdala region, and that voxels contaminated by vessel signal can be easily distinguished from other voxels by their functional connectivity to other voxels containing vessels. In some cases, voxels with some contamination can be seen between uncontaminated voxels and unambiguous BVR voxels, but at some places, BVR voxels and voxels without any visible contamination by venous signals are direct neighbours. In both cases, the distance between unambiguous amygdala voxels and unambiguous BVR voxels is less than 2 mm.

**Figure 6:**
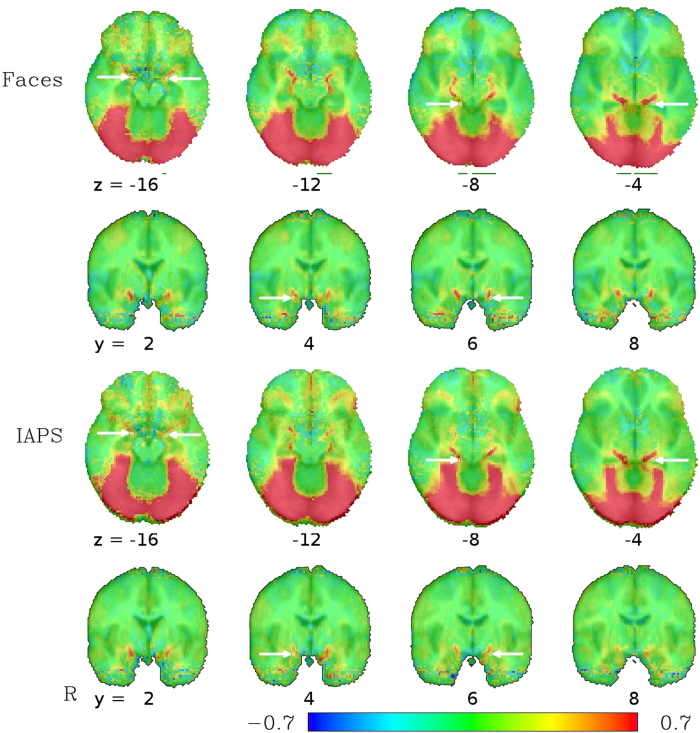
Average functional activation from a dataset of 134 subjects measured with a high-TR (2000 ms) EPI sequence for the contrast between fearful faces (top) and threatening IAPS pictures (bottom) compared to geometric forms in a block-design matching task. As in figure 1, the activation pattern in the amygdala region follows the typical course of the BVR around the brainstem until no longer distinguishable from the activation cluster in the occipital lobe. The values depicted are beta coefficients for the linear models, averaged across subjects, and can be interpreted as percent signal change between the faces/IAPS blocks on one hand and the geometric figures on the other. Arrows depict activation foci from the meta-analysis by Sabatinelli *et al*.^27^, the arrows in the axial slice at z = −16 and in the coronal slices at y = 4 and y = 6 pointing to the amygdala foci, the arrows in the axial slices at z = −8 and z = −4 pointing to the parahippocampal gyrus foci, see table 1. Note the proximity of these foci to the BVR activations for both the amygdala and the parahippocampal gyrus activation foci from the meta-analysis.

**Figure 7:**
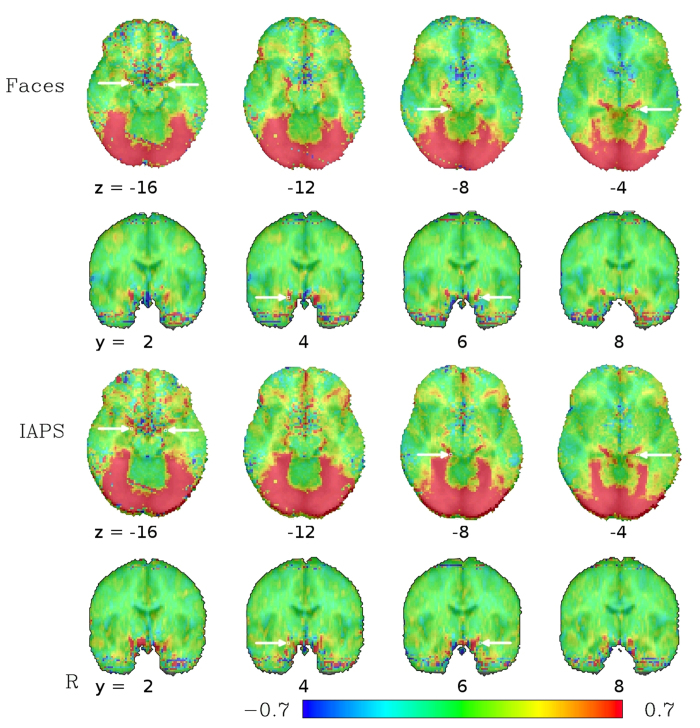
Average functional activation from a subset of 30 subjects of the high-TR (2000 ms) dataset for the contrast between fearful faces (top) and threatening IAPS pictures (bottom) compared to geometric forms in a block-design matching task, similar to figure 6. Activations closely resemble those of that figure despite the smaller sample size. Note that activations in BVR voxels between the amygdala and regions affected by signal loss in proximity of the nasal cavities (characterized by noisy activation patterns) exist as in the complete dataset, but are more difficult to distinguish from the noise near the nasal cavities in the smaller subset.

In the high-TR dataset, using the same preprocessing pipeline as in the low-TR dataset (i.e. standard preprocessing, but without spatial smoothing), similar activation peaks as with the low-TR dataset can be seen. Most important for our purposes is the clear identification of a bilateral linear formation of activated voxels starting around the amygdalae, passing around the brain stem on both sides before converging in the occipital brain and becoming indistinguishable from the large swathes of activation in the visual cortex (see Fig. 6). In the high-TR dataset, the sensitivity in subcortical regions does not allow for an unambiguous identification of the vein at single-subject level, but subgroup analyses reveal that activation in the BVR not only occurs in large sample sizes. Rather, even at the more typical fMRI study sample size of 30 subjects, the activation pattern along the BVR can be clearly discerned (see Fig. 7).

To assess the potential influence of the effects observed in our data on previously published results, coordinates of activation foci for the emotional face > neutral face contrast in the left and right amygdalae, parahippocampal gyri, fusiform gyri and posterior fusiform gyri as provided in the meta-analysis by Sabatinelli *et al*.^27^ (see Table 1 for the coordinates used) were used and compared with our group results (see Figs. 1 and 6). Note the close proximity of the four activation foci identified by the meta-analysis in the ventral brain, designated there as left and right amygdala as well as left and right parahippocampal gyrus, to the course of the BVR as identified in our dataset. The meta-analysis by Fusar-Poli *et al*.^15^ also provides coordinates of peak activation, but in Talairach space. We did not depict them separately in our figures and rather use the coordinates from Sabatinelli *et al*.^27^ to avoid potential errors in the transformation of the coordinates, but it should be noted that after our transformations in MNI space, all but one of the amygdala coordinates provided by Fusar-Poli *et al*.^15^ were within one voxel–2 mm—of the coordinates provided by Sabatinelli *et al*.^27^, indicating that the choice of which meta-analytic coordinates to use did not bias our results. Most, but not all, of the other activation foci identified in the meta-analysis correspond to activation clusters seen in our dataset as well. In particular, the activation foci designated as fusiform gyrus and posterior fusiform gyrus, regions potentially drained by the BVR, correspond to large clusters of activation in our dataset.

Correlations between the signal time courses after accounting for the task blocks show that there are significant connections between the fusiform activation cluster and the ipsilateral BVR cluster, but also between other regions, e.g. between the visual cortex and the fusiform clusters, which might be interpreted as functional connectivity. It is noteworthy though, that the fusiform activation cluster explain more variance in the BVR than the visual cluster (see Table 2). While this is not in itself a proof for a direct connection, it is consistent with the fact that the BVR may drain the fusiform gyrus, but not the occipital lobe.

## Discussion

The increased functional sensitivity of low-TR multiband BOLD EPI made it possible to show that major signal changes measured in the amygdala region in a typical emotional task is not, in fact, located in the amygdala itself. Rather, these signal changes occur in the adjacent Basal Vein of Rosenthal (BVR) that drains large regions of the medial temporal lobe and has confluences from other large veins in the amygdala region, and are therefore largely unrelated to neuronal activity in the amygdala itself. While the suggestion that fMRI being only an indirect measure of neuronal activity is not in the least a novel concept^28^, the possibility of signal changes in veins in the ventral brain at such a large distance from the neuronal origin (in this case, probably the fusiform gyrus) have not been demonstrated before. Although the clarity of the association of this activation locus with the BVR at single-subject level is only achieved using novel low-TR multiband sequences, the impact of its effect on fMRI results cannot be missed even in datasets acquired with more commonly employed EPI sequences and parameters. This can be seen in our comparison dataset as well as in the literature on the mapping of emotion processing as a whole, as exemplified in the comparison of our results with the locations of activation foci from meta-analyses^27^.

As mentioned above, the BVR drains large parts of the medial temporal lobe, but it also usally connects to the deep middle cerebral vein draining the insular and the striatal veins, thereby forming the striatal BVR segment. At the uncus, close to the amygdala, the striatal segment unites with the peduncular BVR segment, where additional peduncular veins join the BVR. The latter often build an anastomosis receiving blood from the contralateral temporal region via the interpeduncular veins. Concerning the peduncular BVR segment the amygdalar vein is an important, but by far not the only, draining vessel of the temporal pole region that variably joins the BVR. Additionally, the venous blood from the anterior region of the medial temporal lobe may not be drained by the BVR only, but also to the superior petrosal or the cavernous sinus^26^. One should thus be careful when making generalizations on the venous structures around the amygdala, as there exist large inter-subject heterogeneity. For example, the anterior segment of the BVR does not or not predominantly drain into the posterior segment and, ultimately, into the vein of Galen, but rather has its own, anterior, drainage, in about a third of the hemispheres—in these cases, the observed activation patterns might differ from those presented in the group average maps in Figs. 1 and 6 in that it would lack the anterior-posterior connection around the brain stem to the vein of Galen. What can be observed rather consistently is the confluence of the aforementioned deep middle cerebral vein, anterior cerebral vein, and the striatal segments in the proximity of the amygdala. Thus, while the course around the brain stem might be one of the most distinctive features of the BVR activation in Figs. 1 and 6, BVR contamination in the amygdala region is likely to occur even in cases where this particular course is not observable. For an excellent schematic of different variants of the BVR, see Fig. 9 by Fernndez-Miranda *et al*.^26^.

Despite this anatomical variability, the confounding effect of the BVR on the signal measured in the amygdala region is very consistent, as it appears in multiple independent datasets under different circumstances. The low-TR multiband dataset using the matching paradigm task had the maximum sensitivity at single subject level due to its high temporal and spatial resolution, the large number of time points acquired and the ability of critically sampling cardiac frequencies at which physiological signal contaminations occur, which could thus be eliminated from the dataset by temporal filtering. Indeed, in this dataset, the systematic stimulus-correlated signal variations are clearly discernable even at the single-subject level (see supplementary video 1) and can be localized to the veins around the amygdala. The resting-state dataset, acquired during the same scan session with the same protocol, confirms that the signal in the voxels that showed highest activation in the amygdala region were characterized by strong correlation to the signal from voxels follwing the course of the BVR further around the brain stem towards the vein of Galen, the most common variant of the draining of the BVR^26^, and from other voxels in regions with major vessels, like the lateral fissure. This pattern contrasts sharply with the pattern of connectivity found in voxels located more clearly within the amygdala itself, which show no significant correlation of their signal with these regions (see Fig. 5). In some cases, voxels partially contaminated by venous signals can be seen between voxels that can be unambiguously attributed to the BVR and the amygdala, and it is noteworthy that there is often less than 2 mm between such unambiguous voxels. This means that when preprocessing pipelines using spatial smoothing with 6 or 8 mm FWHM kernels are applied, as is often the case in standard fMRI preprocessing, the BVR signal contamination is drawn into amygdala voxels that would otherwise have been unaffected. With a smoothing kernel of 8 mm FWHM, for example, this would mean that in a typical amygdala (typically less than 10 mm across), all voxels in the amygdala would be affected by this contamination to some degree. Without spatial smoothing, the spread of the contamination is much reduced, basically to that induced by the point spread function of the MR measurement and partial volume effects, and thus typically reduced to up to the voxel size (typically 2-3 mm).

Still, results based on the low-TR datasets alone might be subjected to criticism concerning the relevance of these results for studies using standard fMRI protocols, and it might be objected that the confounding effect described here is only an artifact of the new measurement protocol used, and the observations made based on this might thus not be broadly applicable to other fMRI studies. This can, however, be ruled out by the comparison with the large dataset using a conventional fMRI protocol, which, when analyzed without spatial smoothing, revealed the same pattern of activation even though it does not share the peculiarities of our multiband acquisition (high sampling rate, large slice gap, etc.). This appearance of the BVR activation in the results in two datasets using very different measurement protocols strongly suggests that its origin is related to the brain rather than the measurement protocol. Both datasets did, however, use the same stimulus, the emotional matching paradigm, and the signal changes in the BVR being correlated to the stimulus blocks imply that they are are related to the brain’s response to the performance of this paradigm in some way.

This discovery has rather wide-ranging implications. Most immediately, it casts doubt on previous findings on amygdala function that rely solely on fMRI as evidence, in particular where reproducibility has been limited and efforts to confirm them have repeatedly proven difficult^8^,^29^. Of course, this should not be interpreted as fMRI being principally unable to detect amygdala activations, and neither do we want to dispute the role of the amygdala in the processing of emotions per se, as this is established well enough even when completely disregarding all fMRI evidence. What can be said, though, is that one should be more wary of fMRI signal changes in this region instead of attributing them to neuronal activity in the amygdala without careful analyses of potential confounding contributions. Due to the confluence of the amygdalar vein into the BVR, it is impossible to say whether—or, to what extent—signal changes measured in BVR voxels may be due to neuronal activity in the amygdala, in more distant brain regions, or both. Furthermore, it is important to acknowledge that, while being a very convenient tool for measuring brain activity ^in vivo^, fMRI also has severe limitations that need to be explored in detail, but are currently often ignored or downplayed^28^.

We chose a broad approach in demonstrating the effects of the BVR in emotional fMRI paradigms to address possible objections that our finding is dependent on the particular measurement technique involved. Indeed, both the cortical activation patterns and the BVR “activations” are remarkably similar between the two different acquisition protocols employed, suggesting that the effect is not merely a peculiarity of one specific acquisition technique. The impact on the wider literature, on the other hand, is more difficult to assess by any means short of a re-analysis of the original data. Still, the robustness and close correspondence of the location of the venous signal between our two datasets together with the fact that the coordinates of activation foci identified in the meta-analysis by Sabatinelli *et al*.^27^ exactly match this location are highly suggestive. Whether a particular finding in the literature reflects amygdala or BVR signal often remains unclear, especially since the figures shown in a paper typically show slices passing through the amygdala, but give no further indication on the extent of activations in other nearby slices that might give more hints at whether the activation pattern continues to follow the BVR. In addition, the ubiquitous preprocessing step of spatial smoothing helps to diffuse BVR signal changes along most of its course except in the immediate vicinity of the amygdala, i.e. in the region of the confluence of the BVR’s striatal into the anterior peduncular segment. There, three-dimensional gaussian kernels centered in different voxels of the BVR overlap in amygdala voxels, thus strengthening the impression that the activation peak arises from the amygdala itself.

The resting-state findings illustrate that the signals seen in the voxels we found activated in the emotional task in the amygdala region are most strongly correlated with signals in areas characterized by large vessels, such as around the brain stem and in the lateral fissure. This highlights that signal changes in these areas are not likely to reflect neuronal activity at the location of the measured signal change, but rather (task-related) vascular effects (possibly related to neuronal activity in more distant brain regions). Nevertheless, since the task activations are correlations of the signal time series with a block design, they should not be thought of as representing physiological pulsations—the effect clearly emerges from the presentation of the stimulus. This also means that regression-based methods as otherwise employed to eliminate physiological effects cannot be used in this context, as the elimination of a stimulus-correlated signal as a nuisance regressor would negate all stimulus effects from later analyses

A further question to ask here is what the mechanisms are that lead to the stimulus-correlated signal changes in these voxels. The most likely candidate seems that it originates from blood drained from other brain regions which are directly activated by the task. Among the regions identified in our datasets, the fusiform gyrus appears to be the region with the largest (both by magnitude and extent) activation observed among the regions which most likely drain to the BVR. The ANOVA results, indicating that the residual time series in the fusiform activation clusters best explain the signal variation in the BVR, are consistent with that hypothesis. It also corroborates the observation by Manuck *et al*.^11^ quoted above that lateralization differences in activations in the amygdala region might be related to differences in visuo-spatial processing demands of the paradigms used, and provides a possible explanation of the mechanisms causing it. Furthermore, a large fMRI study by Mende-Siedlecki *et al*.^30^ including 215 subjects recently identified a network active in facial recognition regardless of emotional content—this network also consisted of the amygdalae and the fusiform gyri, suggesting a connection between the two in their activation to the presentation of pictures of faces.

If this hypothesis on the origin of the signal change is true, it would also suggest that an emotional task widely used to produce activations in the amydala region actually does not seem to involve the amygdala in a way robustly measureable by fMRI. This does not mean that the amygdala is not involved in the processing of this task—as this has been confirmed by multiple studies using different modalities not confounded by venous signal changes^31^,^32^,^33^. However, it might be necessary to design paradigms specifically for fMRI that do not lead to the systematic BVR signal changes that overshadow any neuronal activation in the amygdala region. If the origin of the BVR signal fluctuations lies in the fusiform gyrus, this might not be easy given the ubiquitous involvement of the fusiform in face and object recognition^34^—tasks without visual cues might perhaps be worth experimenting with. Alternatively, fMRI studies aiming to test activations of the amygdala could be combined with a careful work-up of the regional venous drainage, e.g. based on phase contrast angiography.

Beyond the implications for research on the amygdala itself, if the effect indeed originates in the fusiform gyrus, then our results demonstrate that BOLD signal changes induced by neuronal activity can occur in voxels much farther from the actual source of activation than previously believed. It is thus plausible that such effects might also occur in other areas of the brain characterized by the presence of large vessels and where fMRI currently often leads to ambiguous results—the coordinates for the parahippocampal gyrus noted in Sabatinelli *et al*.^27^ also fall on BVR voxels in our analysis, and the insula might also be a worthwile target for similar analyses.

Another intriguing possibility is that the signal changes in the BVR might have no localizable origin at all: in an analysis of task response in the brain under low-noise circumstances and thus high statistical power, Gonzalez-Castillo *et al*.^35^ showed that signal time courses in 95% of brain voxels were correlated to the task, albeit only with a small magnitude of task-related signal change and therefore below the detectability threshold of typical fMRI studies with lower power. Nevertheless, the confluence of blood with task-related oxygenation-level changes from wide areas might lead to a larger net sum effect in the veins draining these regions, and thus to a detectable signal change in the veins despite the signal changes in the individual regions contributing to it being too small to be detected in the experiment. Further research is needed to pinpoint the exact origin and mechanisms of the BVR signal fluctuations and clarify whether a local (e.g., the fusiform gyrus) or a more global origin is more plausible.

While at first glance, the implication that past investigations of emotional processing pathways in the brain have been heavily confounded by a physiological artifact seems largely negative, but the flip side of this coin is that, with current methods, the identification of this artifact can be rather easy. In addition, the robustness of the localization of the vein after normalization in standard space (MNI in our case as well as the meta-analysis by Sabatinelli *et al*.^27^) means that researchers can easily identify the location of their unsmoothed activation peaks in MNI space and find out whether they match the coordinates of the BVR provided here. A more direct and rather conservative approach might be to use voxelwise resting-state connectivity from potentially contaminated voxels and discard voxels based on their connectivity pattern (see Fig. 5) at single-subject level, which might prove a promising method provided that a resting-state scan using the same measurement protocol and slice positioning is available. For group level analyses, in view of anatomical differences between subjects leading to a large number of voxels affected in at least some subjects, this type of method might need some additional refinement, though. We hope that by eliminating this artifact from the data—either by simply discarding affected voxels or perhaps in the future by more sophisticated techniques—a more unambiguous investigation of amygdala functioning using fMRI might lead to more convergent findings in the near future.

## Materials and Methods

### Subjects

Sixteen healthy subjects (9 females/7 males, mean age 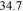, SD 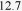) were recruited at Medical University of Vienna. Exclusion criteria were prior psychiatric or neurologic illnesses, as well as the usual exclusion criteria for MR studies. All subjects gave written informed consent prior to the scan and the study was approved by the Ethics Committee of the Medical University of Vienna. All methods were carried out in accordance to the approved guidelines.

### Data Acquisition Protocols

All MRI scans were performed on a 3 Tesla TIM Trio using the standard 32-channel head coil and whole-body gradients (Siemens Medical Solutions, Erlangen, Germany). First, a high-resolution anatomical image was acquired using MPRAGE with 1 × 1 × 1.1 mm^3^ resolution, and 160 sagittal slices (TE/TR = 4.21/2300 ms, flip angle 90°, inversion time 900 ms). Second, BOLD fluctuations at rest were measured with an advanced, low-TR multi-band EPI-sequence^21^ using 1.7 × 1.7 × 2 mm^3^ resolution, 2 mm slice gap (matrix size 128 × 128, 32 axial slices aligned with the AC-PC line, TE/TR = 31/333 ms, flip angle 30°, multiband factor 8, bandwith = 1776 Hz/Pixel) collecting 1200 volumes. Subsequently, the same EPI sequence was used during a commonly employed matching task^36^ designed to activate the amygdala. Subjects were shown triplets of geometric shapes (as neutral stimuli) and of threatening scenes as well as fearful faces (as emotional conditions) presented in alternating blocks of neutral and emotional stimuli. In this task-fMRI experiment, 1420 volumes were acquired. Finally, susceptibility weighted images (SWI) were acquired at 0.6 × 0.6 × 2.0 mm resolution (matrix size 384
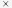
384, 52 slices per slab, 1 slab, TE/TR = 29/42 ms, flip angle 15°) to visualize medium to large venous vessels^37^.

### High-TR Reference Dataset

Furthermore, a second dataset consisting of 134 different healthy subjects (70 f/64 m) was used for comparison purposes. This dataset was also acquired at Medical University of Vienna, and comprised anatomical images using the same MPRAGE sequence as above, as well as functional scans using the same emotional matching task as above, but acquired with a 12 channel head coil and a standard (i.e., non-multiband) single-shot EPI sequence with a TR of 2 s, totalling 280 volumes (TE/TR = 42/2000 ms, 96 × 96 matrix, 210 mm square FOV, 20 axial slices aligned with the AC-PC line, slice thickness = 4 mm, slice gap = 1 mm, interleaved slice acquisition). Earlier results from the same study have been published in Scharinger *et al*.^38^, where a more detailed account of the clinical assessments and inclusion criteria as well as the functional task can be found.

### Preprocessing of Anatomical Data

T1 weighted anatomical images were skull-stripped and normalized to MNI152 space using AFNI, and the transformation matrix of this normalization saved for later use. SWI images were coregistered to the T1 weighted images and subsequently normalized using the transformation matrix from the T1 image normalization.

### Preprocessing of Functional Task Data

Alignment and coregistration of the functional images to the T1 weighted images in MNI space were performed using the AFNI script align_epi_anat.py, using the first volume of each EPI run as the reference volume for the alignment. Functional images were despiked using AFNI 3dDespike, and masked with the binarized skullstripped (using AFNI 3dSkullStrip) T1 weighted image. Voxel time series were converted to percent signal change and bandpassed to frequencies between 0.01 and 0.1 Hz using AFNI 3dBandpass. Voxelwise general linear models were then computed with AFNI 3dDeconvolve, using the motion parameters from the alignment step as covariates and the stimulus blocks convolved with a standard hemodynamic response function as regressors. Finally, for group analyses, the single-subject coefficients for the contrasts ‘Faces – Forms’ and ‘IAPS Pictures – Forms’ were averaged across subjects. The preprocessing steps were applied in the same way to both the high- and low-TR datasets and were chosen to reflect common preprocessing strategies. The only exception to note, however, is the absence of spatial smoothing from the preprocessing pipelines, to avoid the blurring of spatially fine-grained effects.

### Correlation analyses

To assess the signal correlations between the task-activated regions, the residuals from the high-TR general linear models of each subjects were used. Regions of interest defined as the overlap of the group task activation map at a beta value of 0.5 (to avoid having voxels within the mask that have no relationship to the activation observed) with spheres with a radius of 8 mm centered on the ROI coordinates for the left and right amygdalae, parahippocampal gyrus, fusiform gyrus, posterior fusiform gyrus and visual cortex taken from the meta-analysis by Sabatinelli *et al*.^27^, see table 1. Pairwise correlations between these regions were computed, along with linear models incorporating fusiform gyrus, posterior fusiform gyrus and visual cortex as regressors to explain the amygdala signal. Using the latter, analyses of variance were performed to assess the predictive value of these variables on amygdala signals. All of these analyses were performed for the left and right hemispheres separately.

### Preprocessing and Analysis of Resting-State Data

For the resting-state data, preprocessing steps as for the task data were followed, but the bandpassing was extended to a larger band of frequencies, from 0.01 to 0.4 Hz, to harness a larger proportion of the spectrum of the signal time series for the connectivity analyses^22^. For the assessment of BVR functional connectivity, seed time series were extracted as the mean time series from within a mask defined for each subject as the 100 voxels with the highest t-values in its single-subject GLM results for the contrast ‘Faces–Forms’ within in a group mask defined by the intersection of (a) the activation map from the group functional task GLM of the low-TR dataset thresholded at 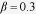 and (b) two spheres with a radius of 14 mm centered on the left and right amygdala. Using this seed, each subject’s whole-brain functional connectivity map of the voxels activated during the task in that particular subject’s GLM was calculated, the correlation coefficients for these maps were converted to z-scores using the Fischer transformation, and the resulting z-maps were averaged across subjects to generate a group connectivity map. In addition, single-subject functional connectivity for individual voxels was assessed using AFNI InstaCorr to evaluate differences in correlation structure seen between individual voxels.

## Author Contributions

R.N.B., E.M.S., B.D., L.P., C.N. and E.M. designed the study. R.N.B., K.K., E.M.S., C.N. were involved in the data acquisition process and R.B., K.K., W.H. and E.M.S. performed the data anaysis. R.N.B., K.K., W.H., C.N. and E.M. wrote the main manuscript text and R.N.B., K.K. and W.H. prepared the figures. All authors reviewed the manuscript.

## Additional Information

**How to cite this article**: Boubela, R. N. *et al*. fMRI measurements of amygdala activation are confounded by stimulus correlated signal fluctuations in nearby veins draining distant brain regions. *Sci. Rep.* **5**, 10499; doi: 10.1038/srep10499 (2015).

## Supplementary Material

Supplementary Video

Supplementary Video Legend

## Figures and Tables

**Table 1:** MNI coordinates of activation foci from the meta analysis on emotional perception by Sabatinelli *et al*.^27^; coordinates are derived from a 100-study meta-analysis on emotional faces >neutral faces contrasts (Table 2 in Sabatinelli *et al.*^27^), except for the lateral occipital cortex coordinates, which are taken from Table 3 in that paper, and originate from a 57-study meta-analysis on emotional scene > neutral scene contrasts.

**x**	**y**	**z**	**Structure**
−20	4	15	R Amygdala
20	6	15	L Amygdala
20	33	4	L Parahippocampal Gyrus
−14	33	7	R Parahippocampal Gyrus
−38	55	20	R Fusiform Gyrus
40	55	22	L Fusiform Gyrus
−38	76	16	R Post. Fusiform Gyrus
40	78	21	L Post. Fusiform Gyrus
−46	68	4	R Lateral Occipital Ctx
48	72	4	L Lateral Occipital Ctx

**Table 2:** Total sum of squares (SSq) of variance in the BVR explained by the fusiform and visual cortex seeds in the residuals of signal time series of the matching paradigm after accounting for the task blocks and other confounds included in the model.

	**Total SSq**	**Visual Cortex SSq**	**Fusiform Gyrus SSq**
Left	10516.6	2695.7 (25.6%)	6349.9 (60.3%)
Right	11040.8	1574.5 (14.2%)	5925.4 (53.7%)

The fusiform gyrus includes both the “fusiform gyrus” and “posterior fusiform gyrus” ROIs from Sabatinelli *et al*.^27^, the visual cortex corresponds to “lateral occipital ctx” there. The p-values for all of the F statistics associated with the given SSq-values are below 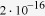.
