# Supplementary material for: fMRI measurements of amygdala activation are confounded by stimulus correlated signal fluctuation in nearby veins draining distant brain regions
Source: Sci Rep. 2015 May 21;5:10499. doi: 10.1038/srep10499 (PMC4440210; doi:10.1038/srep10499)
Supplement: Supplementary Video Legend [file srep10499-s2.pdf]

# fMRI measurements of amygdala activation are confounded by stimulus correlated signal fluctuation in nearby veins draining distant brain regions

Roland N. Boubela[abc†], Klaudius Kalcher[abc†], Wolfgang Huf[abc], Eva-Maria Seidel[d], Birgit Derntl[e], Lukas Pezawas[f], Christian Našel[g], Ewald Moser[abh*]

[a] *Center for Medical Physics and Biomedical Engineering, Medical University of Vienna, Vienna, Austria*
[b] *MR Centre of Excellence, Medical University of Vienna, Vienna, Austria*
[c] *Department of Statistics and Probability Theory, Vienna University of Technology, Vienna, Austria*
[d] *Social, Cognitive and Affective Neuroscience Unit, Department of Basic Psychological Research and Research Methods, Faculty of Psychology, University of Vienna, Vienna, Austria*
[e] *Department of Psychiatry, Psychotherapy and Psychosomatics, RWTH Aachen University, Aachen, Germany*
[f] *Department of Psychiatry and Psychotherapy, Medical University of Vienna, Vienna, Austria*
[g] *Department of Radiology, Tulln Hospital, Karl Landsteiner University of Health Sciences, Tulln, Austria*
[h] *Brain Behaviour Laboratory, Department of Psychiatry, University of Pennsylvania Medical Center, Philadelphia, PA, USA*
[†] *Roland N. Boubela and Klaudius Kalcher contributed equally to this work.*
[*] *Correspondence to ewald.moser@meduniwien.ac.at*

February 26, 2015

**Supplementary Video** Single-subject SWI and Faces-Forms Activation
